# 

**Published:** 1875-07

**Authors:** 


					﻿Vol. 22.
JULY 1875.
No. 7.
TWENTY-SECOND YEAR
HALL’S
Joumal of Health.
** The serial, a portion of which appears in this number, entitled “ Own Stnsant at Mafmcwood,”
has been duly copy-righted according to law.”
PUBLISHED BY THE AMERICAN AND FOREIGN
PUBLICATION CO., 137 EIGHTH ST, NEW YORK.
▲cento for the Trade t
AMERICAN NEWS COMPANY, 121 NASSAU STREET.
NEW YORK NEWS COMPANY, 18 BEEKMAN STREET.
Tense, Twe Dollars a Tesar.
SINGLE NUHMB, 20 CENTS.
				

